# Characterization of proton production and consumption associated with microbial metabolism

**DOI:** 10.1186/1472-6750-10-2

**Published:** 2010-01-20

**Authors:** Karthikeyan Srinivasan, Radhakrishnan Mahadevan

**Affiliations:** 1 Department of Chemical Engineering and Applied Chemistry, University of Toronto, Toronto, Ontario, M5S3E5, Canada; 2 Institute of Biomaterials and Biomedical Engineering, University of Toronto, Ontario, M5S3G9, Canada

## Abstract

**Background:**

Production or consumption of protons in growth medium during microbial metabolism plays an important role in determining the pH of the environment. Such pH changes resulting from microbial metabolism may influence the geochemical speciation of many elements in subsurface environments. Protons produced or consumed during microbial growth were measured by determining the amount of acid or base added in a 5 L batch bioreactor equipped with pH control for different species including *Escherichia coli*, *Geobacter sulfurreducens*, and *Geobacter metallireducens*.

**Results:**

An *in silico *model was used to predict the proton secretion or consumption rates and the results were compared with the data. The data was found to confirm predictions of proton consumption during aerobic growth of *E. coli *with acetate as the carbon source. However, in contrast to proton consumption observed during aerobic growth of *E. coli *with acetate, proton secretion was observed during growth of *Geobacter *species with acetate as the donor and Fe(III) as the extracellular electron acceptor.

**Conclusions:**

In this study, we have also shown that the final pH of the medium can be either acidic or basic depending on the choice of the electron acceptor for the same electron donor. In all cases, the *in silico *model could predict qualitatively the proton production/consumption rates obtained from the experimental data. Therefore, measurements of pH equivalents generated or consumed during growth can help characterize the microbial physiology further and can be valuable for optimizing practical applications such as microbial fuel cells, where growth associated pH changes can limit current generation rates.

## Background

*Geobacter *species are well known for their metal reducing capabilities and are responsible for accelerating the bioremediation of radioactive and toxic metals in subsurface environments [1-3]. They are capable of anaerobic oxidation of organic contaminants with concomitant reduction of metals such as Fe(III) and other contaminants such as Uranium, and consequently are important for groundwater bioremediation [4-7]. Furthermore, *Geobacter *species are capable of direct electron transfer to an electrode motivating their use in microbial fuel cells [8]. The pure culture model of *Geobacter sulfurreducens *from *Geobacteraceae *family has been extensively studied in order to characterize its physiology and to determine the mechanisms associated with the extra-cellular electron transfer. Recently, several genome-wide studies have characterized the unique metabolic features of *Geobacteraceae *including the development of a genome-scale metabolic model of *Geobacter sulfurreducens*, the chemotaxis towards iron, and the synthesis of conductive pili [9-13]. The genome-based modeling revealed that, global proton balance was significantly different for *Geobacter *species, which rely on extra-cellular electron transfer to insoluble substrates, in comparison to aerobic organisms which reduce oxygen to water. Furthermore, improved understanding of the global proton balance in *Geobacter *species can provide insights on the physiology of other species capable of extra-cellular electron transfer such as *Rhodoferax*, *Shewanella *regardless of the mechanism of the electron transfer since protons are not consumed at the terminal electron accepting step in all cases.

Changes in the environmental conditions such as pH, temperature, osmolarity, and electron donor availability can alter the trans-membrane pH gradient, total proton motive force and affect the internal pH of the cells as well as the energetics [14]. Hence, in order to maintain energy homeostasis, cells have to actively regulate the internal pH by secreting or consuming protons. The medium can act as a source/sink for protons depending on the substrates that are present. The characterization of this proton exchange can provide additional insights on the metabolism and physiology of less studied organisms such as *Geobacteraceae *that use extra-cellular electron transfer for energy generation.

Several *in silico *models have been constructed to describe and predict the intracellular metabolism at the genome-scale for many organisms including *E. coli, S. cerevisiae*, and *B. subtilis *[15-20]. Specifically, the constraint-based modeling approach has been used to study *E. coli *metabolism for over ten years [21,22]. These constraint-based models provide a framework to predict cellular physiology including growth and by-product formation across a range of growth environments [23]. Predictions of proton secretion/consumptions rates associated with *E. coli *grown under aerobic conditions in the presence of varying electron donors has been reported [24]. Consequently, the measurements of the proton secretion/consumption associated with the cellular growth and metabolism can be used to derive additional information on physiology that can be used to further validate the models.

The *in silico *analysis of *Geobacter sulfurreducens *metabolism revealed that differences in global intracellular proton balance can lead to lowered biomass yields during growth with extracellular electron acceptors such as Fe(III), relative to the growth with electron acceptors reduced in the cytosol, such as fumarate [25], highlighting the need for detailed analysis of the proton exchange associated with metabolism. One of the major concerns during high-density growth of *E. coli *on excess glucose under aerobic conditions is the formation of acidic by-products [26-29]. Several studies have been carried out to determine the effect of fermentation conditions on accumulation of acetate and other by-products [26,30-33].

**Figure 1:**
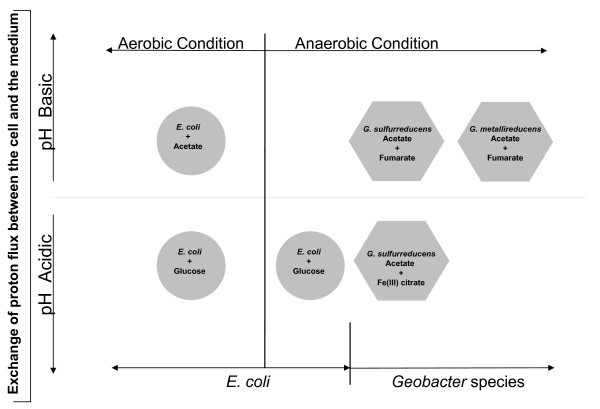
**Influence of proton flux on pH of the medium for cultures grown under aerobic and anaerobic conditions**.

The bacterial cells grown in complex environment use the available substrate either preferentially or simultaneously depending on the growth condition. The metabolism of glucose and acetate in *E. coli *has been extensively investigated during the past 50 years [34] and are well characterized. Although dynamic models of growth on mixed substrates have been developed based on experimental data from substrate uptake rate and biomass production measurements [35-37], no previous experimental study, to our knowledge, measured the net production or consumption of protons in different environments, even though in the case of *E. coli*, modeling studies suggested that the direction of proton exchange was found to depend on the electron donor [24]. The scope of this work was to first confirm the previous model predictions of proton exchange for *E. coli *and subsequently, to measure the proton production or consumption during growth of *Geobacter *species on acetate with fumarate or ferric citrate and compare it with the *in silico *model predictions (Figure 1). Models that can predict the rate of proton secretion or consumption under a wide range of environmental conditions will be valuable in developing model-based approaches to the optimization of power production in microbial fuel cells and the *in situ *bioremediation of contaminated subsurface environments, where the effect of pH changes is critical.

## Results

It has been shown previously that for *Geobacter *species, global proton balance has been shown to be critical in determining biomass yields during the respiration of extracellular electron acceptors. Here, we have investigated the relation between the growth environment and proton generation/consumption for *E. coli*, *G. sulfurreducens *and *G. metallireducens*. Specifically, we studied the variation in proton exchange as a function of the electron donor (glucose, acetate) for *E. coli *and the electron acceptor (fumarate/Fe(III) citrate) for *G. sulfurreducens *and *G. metallireducens*. The results from these experiments are detailed in the following sections.

### 2.1 Analysis of Proton Production in *E. coli*

**Figure 2:**
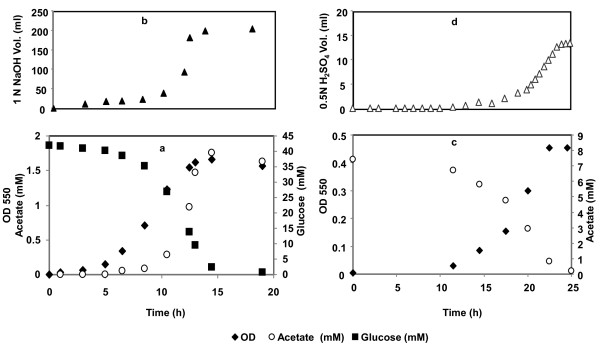
**Batch culture of *E. coli *W3110 with pH Control in a bioreactor in the absence of buffer**. (a) growth with glucose; (b) base added; (c) growth with acetate; (d) acid added.

Recent genome-scale metabolic models incorporate detailed charge and elemental balance, and consequently, the number of protons consumed or generated for every metabolic reaction is represented in the model. This feature allows the calculation of proton flux generated or consumed during growth in varied environments. Reed *et al*. [24] described the variation of proton secretion flux with the carbon source. Depending on the limiting substrate, the exchange of protons across the membrane can produce either basic or acid environment. For *E. coli*, during growth with acetate as electron donor, the external medium environment became basic and acid was added to maintain neutral pH as shown in Figure 2 (panels c & d). The maximum observed OD_550 nm _value was 0.45 and substrate was completely consumed after 25 hrs. Similar experiment was carried out for growth of *E. coli *on glucose. However, in the case of growth with glucose, base was added in order to maintain circumneutral pH (Figures 2a &2b). The variations in proton generation with the electron donor are also consistent with the reduced nature of glucose as compared to acetate. Thus, glucose oxidation resulted in generation of excess protons that are not consumed completely by the formation of water during the oxygen reduction. These excess protons are secreted into the medium along with other acid by-products resulting in an acidification of the medium forcing the addition of base to maintain a constant pH. Table S1 (Additional file 1) shows the initial and final biomass concentrations and the amount of acid/base added for all of the studies in this report.

### 2.2 Proton Production in *Geobacter *Species

*G. sulfurreducens *and *G. metallireducens *containing the fumarate transporter [38], were grown in a batch bioreactor, in the absence of a buffer, with acetate (10 mM) as electron donor and fumarate (40 mM) as the sole electron acceptor as previously described [39]. In control experiment(s), the inoculum(s) was omitted to investigate changes in medium pH. The medium pH remained neutral and there was no addition of either acid or base in all control experiments (data not shown).

**Figure 3:**
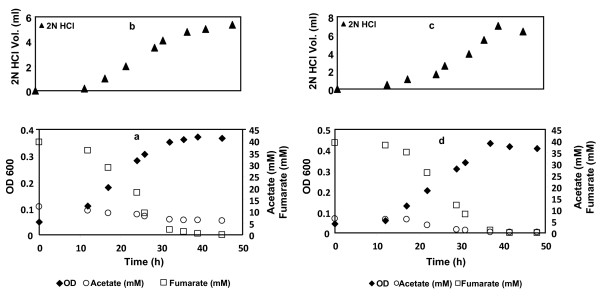
**Proton consumption by *Geobacter *species in the absence of bicarbonate with pH control**. Batch culture of *G. sulfurreducens *with acetate and fumarate (a); acid added (b); batch culture of *G. metallireducens *(c); acid added (d).

**Figure 4:**
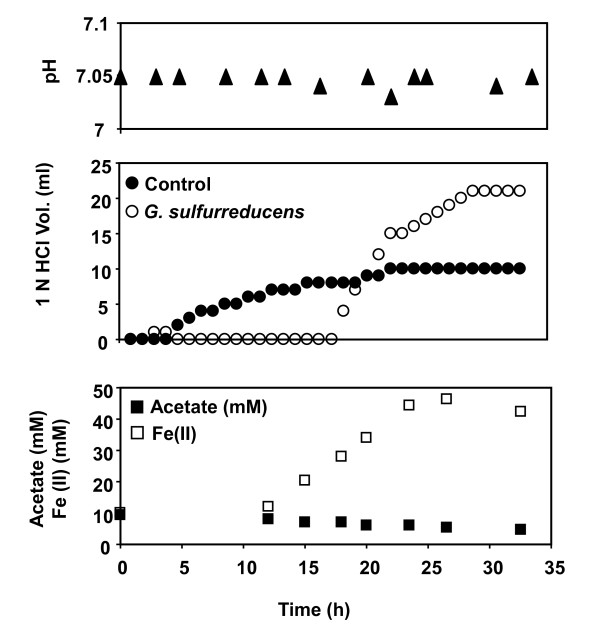
**Proton production by *Geobacter sulfurreducens *in the absence of bicarbonate with acetate and ferric citrate**.

Both the *Geobacter *strains showed a similar trend in the amount of acid added as compared to the aerobic growth of *E. coli *with acetate (Figure 3). The pH of the culture medium gradually increased and acid was added to maintain neutral pH as shown in Figures 3b &3c. *G. sulfurreducens *and *G. metallireducens *obtained a maximum OD value of 0.37 and 0.42 respectively. Reed *et al*[21,24] showed that by choosing different electron donors, the medium can be made either acidic or basic. In addition to the variation of the electron donors, we have also investigated the influence of electron acceptors on the medium pH. Figure 4 shows the acetate consumption by *G. sulfurreducens *during growth in the presence of acetate (10 mM) and Fe(III) citrate (40 mM).

During the growth of *G. sulfurreducens *with Fe(III) citrate as sole electron acceptor, protons were produced by the cells. The cell growth was inferred indirectly by acetate oxidation and Fe(III) reduction since Optical Density (OD) measurements are not possible and cell counts lead to significant variances in the measurements. In control experiments, the acid addition started at the beginning and continued for 25 hrs until volume of 10 ml was dispensed into the bioreactor to maintain neutral pH (7.0), however reduction of Fe(III) was not observed. This observed increase in pH of the uninoculated medium upon N_2 _sparging can be explained by an inorganic reaction equilibria model (data not shown). In contrast, during the growth of *Geobacter sulfurreducens*, the medium was maintained at neutral pH without external addition of either acid or base. This can be attributed to the secretion of protons during the growth phase *G. sulfurreducens *as a result of cellular metabolism.

However, as Fe(III) was depleted at 25 hr, the cells reached stationary phase, consequently proton production associated with growth stopped and the acid had to be gradually added externally by the pump until it reached volume of 21 ml. The change in pH of the medium containing Fe(III) citrate in control experiments could be attributed to the potential release of hydroxyl ions from inorganic equilibrium reactions. The hydroxyl ions are reactive species, and will react immediately to form Fe(OH)_3 _which can precipitate in the solution. Similar results were reported by Francis and Dodge [40].

**Figure 5:**
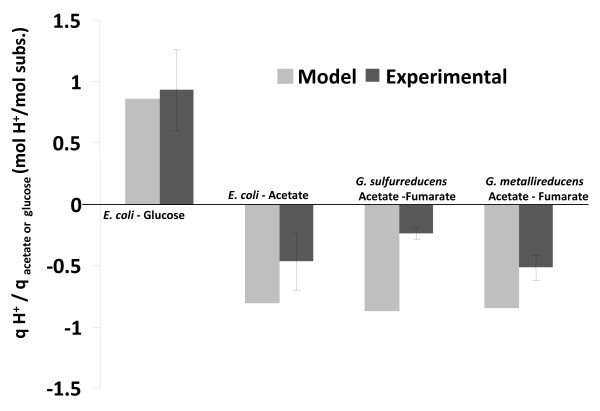
**Comparison of model predictions of proton secretion flux with experimental data**. Positive flux corresponds to import and negative flux corresponds to export of protons for *E. coli *and *Geobacter *species.

This study shows that proton exchange also depends on the choice of the electron acceptor. Specifically, for the case of the extracellular acceptors such as Fe(III), there appears to be net production of protons. This result is consistent with the model predictions of proton secretion during Fe(III) reduction [25] and provides additional evidence to support the hypothesis, that the biomass yield of *G. sulfurreducens *in the presence of Fe(III) citrate as the electron acceptor is lower than fumarate due to the need to maintain global proton balance by exporting protons from the cell at the expense of ATP generation. The comparison of predicted and measured proton fluxes is presented in Figure 5. Here, the positive proton flux represents secretion and negative flux represents uptake. The model predictions of proton production are consistent with the data and are within the experimental error for *E. coli *cultures, where as for the *Geobacter *species the model predictions qualitatively predict proton consumption although the extent of the predicted proton consumption is higher than what is observed experimentally.

## Discussion

Bacterial cells have to regulate the cytoplasmic pH to survive in the constantly changing environment as bacterial growth is dependent on substrate availability, as well as the redox potential and the pH of the medium. The regulation of internal pH involves proton export that requires energy in the form of ATP. The biochemical reactions in the cytoplasm associated with metabolism can lead to a net proton production or consumption. The variations in proton concentration in the cytoplasm during growth, can affect the kinetics and the thermodynamic feasibility of biochemical reactions necessitating active regulation of pH. The energetic cost of pH regulation via exporting protons associated with metabolism can impact the biomass yield of the organism and the extent of this effect can vary with the environment.

For example, in *E. coli *grown with glucose as the electron donor, organic acids released during growth greatly contribute to changes in pH of the medium. Previous studies [41-43] have shown that changes in pH of the culture medium affects the growth of microorganism. However, measurement of such pH changes can be used to analyze the metabolic state of the cells. Previously, changes in pH have been used to identify the onset of product synthesis using on-line parameter estimation in fermentation process San *et al *[44]. More recently, the *in silico *model by Reed *et al *[24] predicted the variation of proton secretion flux for *E*. *coli *with different electron donors. In this study, we have shown that the *in silico *model predictions were consistent with experimentally observed proton production or consumption in 1) cultures of *E. coli *grown aerobically on different electron donors, and 2) *Geobacter *species grown anaerobically on acetate with different electron acceptors.

During aerobic growth, intracellular protons along with electrons transferred from the electron transport chain combine with oxygen resulting in the formation of water as well as removal of intracellular protons. In contrast, during anaerobic growth with metals, the protons generated during substrate oxidation cannot be removed at the terminal electron accepting step. Consequently, proton generation profile during anaerobic growth will be markedly different from aerobic growth in similar conditions. In *Geobacter *species, acetate oxidation is coupled with reduction of fumarate which is the terminal electron acceptor. Biomass yield of *G. sulfurreducens *during acetate oxidation with fumarate as the electron acceptor is higher than the corresponding yield during Fe(III) reduction. This has been attributed to the differences in proton generation associated with metabolism, since during Fe(III) reduction, protons are generated and there is a net proton efflux that consumes energy. During the reduction of soluble acceptors such as fumarate, protons are consumed at the terminal electron accepting step resulting in a net proton uptake. In this study, during Fe(III) reduction, we observed a net production of protons, whereas, we found that during fumarate reduction acid was added to maintain pH balance indicating proton consumption.

The predicted proton uptake agreed with the measured acid addition for *E. coli *grown on glucose. However, for all other cases model predicted higher proton consumption by the cells. One reason for this discrepancy could be the incorrect representation of protons that are consumed during biomass production. Another factor could be either the bicarbonate produced during growth of *E. coli *that can buffer some of the pH changes experimentally or the production of organic acids that are not represented in the model. Yet another factor could be the uptake of NH_4_^+ ^followed by proton extrusion observed for bacteria and fungi [45-47]. While these mechanisms might explain the discrepancies for *E. coli*, it is important to note that CO_2 _is also consumed by *G. sulfurreducens *and acids cannot be secreted during acetate oxidation. Hence, the factors that contribute to proton production during growth of *Geobacter *species needs to be further elucidated. These effects are not incorporated in the current stoichiometric model which does not include the acid/base equilibrium reactions in the reactor. In addition, since the proton exchange flux was calculated based on the acid/base added during batch growth experiments, errors in the measurements could also lead to additional discrepancy between the model and the data.

## Conclusion

The results in this study clearly highlight the ability of the genome-scale models that incorporate detailed proton balanced biochemical reactions to predict, albeit qualitatively, the proton exchange rates and other aspects of physiology. Future research in this area would extend the scope of genome-scale models by including detailed inorganic equilibria reactions to accurately represent the abiotic reactions that can consume or produce protons in the medium. In order to effectively incorporate the abiotic reactions in the model, additional experimental measurements such as the CO_2 _production/consumption rate along with NH_4 _consumption rate will be required. Such an integrated description will be valuable in practical applications such as the in situ bioremediation or microbial fuel cells, where changes in environmental pH would greatly affect the respiration and metabolism. Therefore, models that can predict the rate of proton secretion or consumption under a wide range of environmental conditions will be valuable in predicting the growth physiology as well as the extent of respiration and for prioritizing strategies for bioremediation of contaminated subsurface environments and optimization of power production in microbial fuel cells.

## Methods

### 5.1 Strains and Medium Composition

The bacterial strains used were *E. coli *W3110, *G. sulfurreducens *(DL1), and *G. metallireducens*. The cultures of *E. coli *were grown on a defined medium containing mineral salts as described by Causey *et al*[48] and maintained at 37°C. *G. sulfurreducens *and *G. metallireducens *were cultivated anaerobically at 30°C in a freshwater fumarate medium as previously described by Esteve-Nunez *et al*[39]

### 5.2 Batch Culture

A fully instrumented Minifors bioreactor was used for fermentation. The impeller speed was maintained constant at 200 RPM. The temperature was maintained at 30°C and 37°C for *Geobacter *strains and *E. coli *respectively. The pH was adjusted to 7.0 by automatic addition of equimolar solution of 2N HCL or 0.5N H_2_SO_4 _or 1 M NaOH. The electron donor for *Geobacter *species was acetate, supplied in the form of sodium acetate (10 mM). The culture media was constantly sparged with 100% Nitrogen for *Geobacter *strains and compressed air for *E. coli *respectively. For *E. coli*, either glucose (50 mM) or acetate (10 mM) was used as substrate. The working volume of the bioreactor for cultivation was 2.2L. In both cases, the phosphate and carbonate salts that provide buffering capacity was not included in the medium as the pH in the bioreactor was being controlled through the addition of acid or base via an external feedback control loop. The inoculum for batch cultivation was prepared in shake flask by growing the culture either on glucose or acetate for *E. coli *under aerobic conditions. *Geobacter *strain(s) were grown in a stationary incubator at 30°C. Culture samples were taken at regular time intervals and stored at -20°C for HPLC analysis.

### 5.3 Analytical Methods

The concentrations of organic acids and glucose in the culture samples was measured by Dionex HPLC equipped with UV detector, Refractive Index (210 nm) and Bio-Rad Aminex HPX-87H column. The cell growth was monitored by optical density measurements, using a spectrophotometer at OD_550 nm _and OD_600 nm _for *E. coli *and *Geobacter *species respectively. The cell mass is estimated for *E. coli *as 1.0 OD_550 nm _is equivalent to 0.33 g dry cell weight/litre [48] and for *Geobacter *as 1.0 OD_600 _is equivalent to 0.47 g dry cell weight/litre. Fe(III) reduction was monitored by measuring the amount of Fe(II) formed using the Ferrozine assay as reported in [4].

### 5.4 Simulations

The experimental values were compared with the *in silico *models. For *E. coli*, the results were compared with the model iJR904 predictions using the COBRA tool box described by Becker *et al*. [49], while for the *Geobacter *species, the model presented in Mahadevan *et al*. [25] and Sun *et al*. [50] was used to simulate the metabolism. All simulations were carried out using the constraint-based modeling approach described in Becker *et al*. [49].

In this approach, the known biochemical reactions are inferred from the genome annotation and assembled into a genome-scale metabolic network. The stoichiometry of resulting reaction list is represented by a matrix (S), whose columns correspond to the reactions in the network and the rows correspond to the metabolites in the network. A set of linear constraints relating the fluxes (v) are derived based on the assumption that the metabolite pools have to be balanced during cell growth as there is no net production or consumption of metabolites. These linear equations derived from the genome-scale network are typically undetermined as there are more variables than equations resulting in plurality of solutions for the flux through the metabolic network. Hence, in order to determine a sole flux vector, a linear optimization problem to maximize a cellular objective such as the growth rate (represented by the objective weight vector, c) in the presence of bound constraints on the fluxes (v) is formulated as show below.

### 5.5 Determination of specific rate of proton exchange (qH^+^)

The pH in the bioreactor is maintained constant by addition of acid or base. Therefore, one can calculate the total amount of protons consumed or produced in the medium from the total amount of acid or base added. If protons are consumed as a result of cellular metabolism, then the pH in the medium will increase as the proton concentration decreases and on the contrary, if protons are secreted into the medium as a result of metabolism, the pH in the medium will decrease as proton concentration increases. In the absence of a buffer, the net protons produced or consumed can then be related to the rate of production using the following equation

Where X is Biomass Concentration (gdw/l), N is Normality of base or acid added, V is Volume of Medium (l), F is the flow rate of acid or base added (l/hr), and q_H _is the proton exchange rate (mol/gdw hr). Since X varies with time, even though the pH is constant, we first obtain the proton yield during the growth phase rather than the absolute rate of proton production (q_H_).

During balanced growth, the rate of proton production is directly proportional to specific growth rate described by Larrson *et al*. [51] and Ayaaki *et al*. [52]

Where Y_X/H _is the yield of protons produced per gram of biomass (moles/gdw), ΔX is the change in biomass concentration, t_o_, t_f _are the times corresponding to the beginning and the end of the growth phase.

## Authors' contributions

KS designed and performed the experiments, interpreted data and wrote the paper, RM conceived of the study, interpreted data and wrote the paper. All authors read and approved the final manuscript.

## Supplementary Material

Additional file 1 **Table S1**. Initial and final biomass concentrations and the amount of acid/base added. Click here for file
