# Supplementary material for: Characterization of proton production and consumption associated with microbial metabolism
Source: BMC Biotechnol. 2010 Jan 20;10:2. doi: 10.1186/1472-6750-10-2 (PMC2831035; doi:10.1186/1472-6750-10-2)
Supplement: Additional file 1 — Table S1. Initial and final biomass concentrations and the amount of acid/base added. [file 1472-6750-10-2-S1.PPT]

## Slide 1
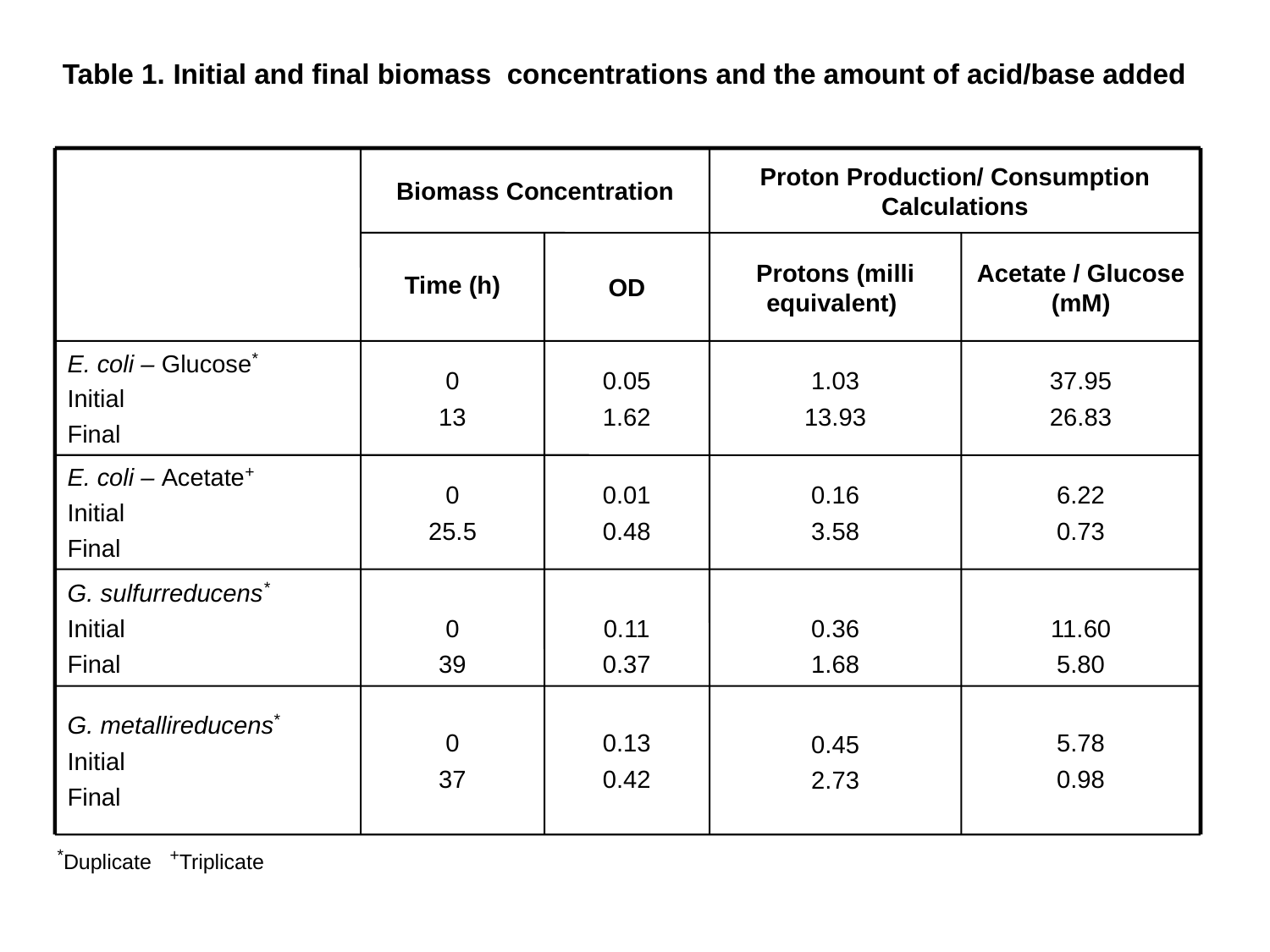

Table 1. Initial and final biomass concentrations and the amount of acid/base added
Biomass Concentration
Proton Production/ Consumption Calculations
Time (h)
OD
Protons (milli equivalent)
Acetate / Glucose (mM)
E. coli – Glucose*
Initial
Final
0
13
0.05
1.62
1.03
13.93
37.95
26.83
E. coli – Acetate+
Initial
Final
0
25.5
0.01
0.48
0.16
3.58
6.22
0.73
G. sulfurreducens*
Initial
Final
0
39
0.11
0.37
0.36
1.68
11.60
5.80
G. metallireducens*
Initial
Final
0
37
0.13
0.42
0.45
2.73
5.78
0.98
*Duplicate +Triplicate
